# Brain-Derived Neurotrophic Factor Is an Important Therapeutic Factor in Mesenchymal Stem Cell Secretions for Treatment of Traumatic Peripheral Pelvic Injuries

**DOI:** 10.3389/fncel.2022.866094

**Published:** 2022-05-18

**Authors:** Xiaoyi Yuan, Brian M. Balog, Dan Li Lin, Brett Hanzlicek, Mei Kuang, Hao Yan, Steve J. A. Majerus, Margot S. Damaser

**Affiliations:** ^1^Department of Biomedical Engineering, Lerner Research Institute, Cleveland Clinic, Cleveland, OH, United States; ^2^Advanced Platform Technology Center, Louis Stokes Cleveland Veterans Affairs Medical Center, Cleveland, OH, United States; ^3^Department of Urology, Tongji Hospital, Huazhong University of Science and Technology, Wuhan, China; ^4^Department of Biology, University of Akron, Akron, OH, United States; ^5^Department of Urology, Xuanwu Hospital, Capital Medical University, Beijing, China; ^6^Glickman Urological and Kidney Institute, Cleveland Clinic, Cleveland, OH, United States

**Keywords:** siRNA knockdown, leak point pressure, neuromuscular junctions, reinnervation, urethra, external urethral sphincter, neuroregeneration, pudendal nerve

## Abstract

Traumatic neuromuscular injury to the pudendal nerve and urethra during childbirth does not regenerate well and contributes to stress urinary incontinence in women. Mesenchymal stem cells (MSCs) can improve neuroregeneration *via* their secretions, or secretome, which includes brain-derived neurotrophic factor (BDNF). In this study, we investigated whether BDNF is a key factor in the secretome of MSCs for the facilitation of functional recovery following a dual simulated childbirth injury. BDNF knockdown (KD) MSCs were created using an anti-BDNF shRNA lentivirus vector. A scrambled sequence was used as a transduction control (scrambled). Cells were cultured for 24 h before media was concentrated 50x to create concentrated conditioned media (CCM) containing MSC secretome. CCM of unmanipulated MSCs was screened for high BDNF expression (high BDNF CCM). Concentrated control media (CM) was created by concentrating media not conditioned by cells. Female Sprague-Dawley rats underwent bilateral pudendal nerve crush and vaginal distension (Injury) or sham injury. One hour and 1 week after injury, sham injured rats received CM, and injured rats received CM, high BDNF CCM, KD CCM, or scrambled CCM (300 μl intraperitoneally). Three weeks after injury, rats underwent leak point pressure (LPP) and pudendal nerve sensory branch potential (PNSBP) recordings. The urethra and pudendal nerve were harvested for anatomical assessment. ANOVA followed by the Student-Newman-Keuls test determined significant differences between groups (*p* < 0.05). BDNF KD CCM had significantly decreased BDNF concentration compared to scrambled CCM, while the concentration in high BDNF CCM was significantly increased. LPP was significantly decreased in CM and KD CCM treated animals compared to sham injury, but not with scrambled or high BDNF CCM. PNSBP firing rate showed a significant decrease with CM treatment compared to sham injury. Neuromuscular junctions in the urethral sphincter in KD CCM, scrambled CCM, and high BDNF CCM were healthier than CM treated rats. While anatomical and nerve function tests demonstrate regeneration of the pudendal nerve with any CCM treatment, LPP results suggest it takes longer to recover continence with reduced BDNF in CCM. BDNF in MSC CCM is an important factor for the acceleration of recovery from a dual nerve and muscle injury.

## Introduction

Traumatic injuries can be caused by automobile or industrial accidents, violence, and combat injuries; however, while not typically characterized as traumatic, childbirth also causes damage to nerves, muscles, and the supportive tissues of the pelvic floor (Noble et al., 1998; Gray, 2004; Taylor et al., 2008). Traumatic injuries lead to loss of motor and sensory function and potentially the development of neuropathic pain (Chan et al., 2014). Since few patients achieve full recovery, patients will live the rest of their lives with these symptoms, affecting many patients in the prime of their lives (Karsy et al., 2019).

While modern medicine has helped save maternal and newborn lives in childbirth, damage to the lower urinary tract and pelvic floor during delivery can lead to stress urinary incontinence (SUI), the unwanted leakage of urine from increased abdominal pressure (Meyer et al., 1993; Abrams et al., 2002; Gray, 2004; Salam et al., 2014; Ng et al., 2017; Daly et al., 2018). Women suffering from post-partum SUI are 2.5× more likely to develop it later in life, suggesting insufficient regeneration after childbirth as a contributor to SUI (Meyer et al., 1993; Eftekhar et al., 2006).

One of the critical components of urethral closure pressure and maintenance of continence is the external urethral sphincter (EUS; Delancey et al., 2008). The EUS is innervated by the pudendal nerve (PN) and both can be injured as the baby’s head passes through the birth canal during delivery (Snooks et al., 1984, 1986; Meyer et al., 1993). It has been theorized that this combinational neuromuscular injury contributes to the pathophysiology of SUI (Swash, 1990).

Preclinical rodent studies of simulated childbirth injuries have shown that the combination of vaginal distension (VD), which injures the EUS, and a PN crush (PNC), significantly delays recovery of urinary continence (Jiang et al., 2009a, b). Additionally, while VD does not itself reduce PN function when VD is added to PNC, it significantly delays PN functional recovery (Jiang et al., 2009a, b). This delay in functional recovery is supported by inadequate expression of brain-derived neurotrophic factor (BDNF) by the EUS after dual injury (Pan et al., 2009). However, administration of BDNF or electrical stimulation of the PN in turn accelerates functional recovery after injury (Gill et al., 2013a; Jiang et al., 2013). The above facts suggest that BDNF may be a key factor for PN regeneration.

Mesenchymal stem cells (MSCs) have great regenerative potential, primarily *via* their secretions, which include growth factors, cytokines, and extracellular vehicles (Zhang et al., 2007; Barzilay et al., 2009; Mias et al., 2009; Penn, 2012; Peter et al., 2012; Tran and Damaser, 2015). We have previously demonstrated that MSCs or their secretions, termed the secretome, accelerate functional recovery after a dual simulated childbirth injury consisting of PNC and VD (Deng et al., 2015; Janssen et al., 2019). The aim of this study was to investigate whether BDNF is a critical factor in the secretome of MSCs for the facilitation of functional recovery following a dual neuromuscular simulated childbirth injury. We hypothesized that BDNF is necessary to facilitate functional regeneration *via* the secretome of MSCs.

## Material and Methods

### Study Design

**Figure 1:**
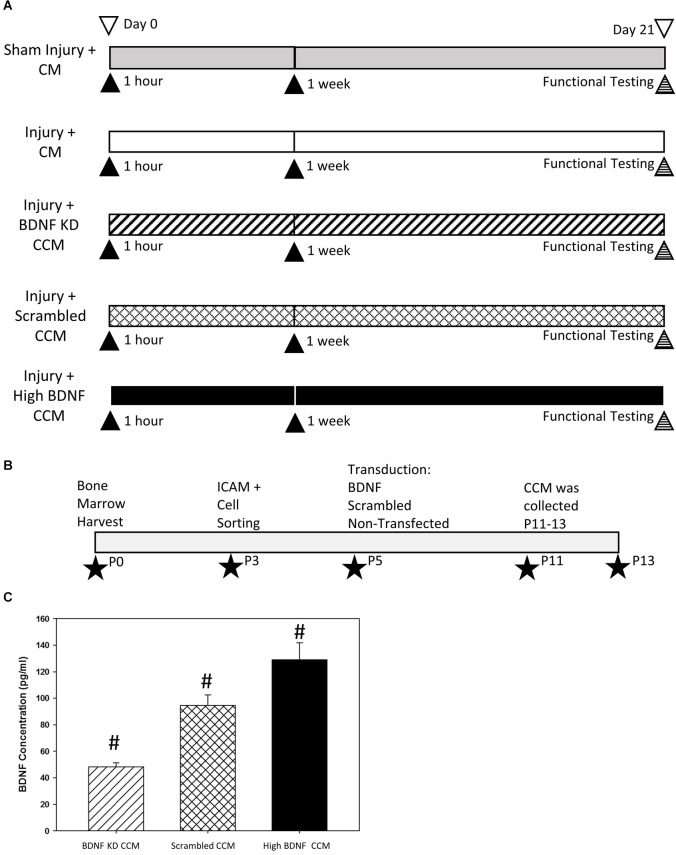
Experimental design diagrams and BDNF knockdown validation. Experimental design **(A)** indicating the five experimental groups: Sham Injury + Control Media (CM), Injury (Pudendal Nerve Crush + Vaginal Distension) + CM, Injury + Brain-Derived Neurotrophic Factor Knockdown (BDNF KD) Concentrated Conditioned Media (CCM), Injury + Scrambled CCM, or Injury + High BDNF CCM. ^▴^Indicates an intraperitoneal injection of treatment. Experimental timeline of mesenchymal stem cell (MSC) purification, transductions, and CCM production **(B)**. ^★^Indicates the passage at which the procedure was performed. Validation of BDNF concentration **(C)**. CCM BDNF concentration. Bars are mean ± standard error of the means from 3 to 5 samples per group. ANOVA followed by a Student-Newman-Keuls *post-hoc* test was used to indicate significant differences. ^#^Indicates a significant difference compared to the other two groups.

This research was approved by the Institutional Animal Care and Use Committee of the Cleveland Louis Stokes Veterans Affairs Medical Center. Sixty-six female, adult, age-matched virgin Sprague-Dawley rats (Envigo, Indianapolis, IN, USA, 226–283 g) were randomly divided into five groups (Figure 1A). The first group underwent sham injury and received concentrated control media (CM; Sham Injury + CM; *n* = 17). The remaining groups underwent dual injury to create SUI and received either one of four treatments: CM (Injury + CM; *n* = 16), concentrated conditioned media (CCM) from MSCs in which BDNF had been knocked down by small interfering RNA (siRNA) transduction (Injury + BDNF KD CCM; *n* = 12), CCM from MSC with scrambled siRNA transduction (Injury + scrambled CCM; *n* = 11), or CCM from unmanipulated MSCs selected for their high BDNF concentrations (Injury + high BDNF CCM; *n* = 10). Each animal received two 300 μl treatments *via* intraperitoneal (i.p.) injections, 1 h after the injury, and 1 week later. All rats underwent functional testing 3 weeks after injury, including leak point pressure (LPP) with simultaneous EUS electromyography (EMG), as well as PN sensory branch potential (PNSBP) recording. Rats were then euthanized and the pudendal nerve and urethra were dissected for anatomical assessment of pudendal nerve regeneration *via* immunofluorescence.

### Mesenchymal Stem Cells (MSCs)

Bone marrow was collected from femurs and tibias of Sprague Dawley female donor rats to obtain MSCs as described previously (Dissaranan et al., 2014). Cells were cultured in normoxic conditions with 5% CO_2_ at 37°C in Dulbecco’s modified Eagle’s low glucose (DMEM) media supplemented with 10% and 12.5% fetal bovine serum (FBS). MSCs were selected by sorting the cells at passage 3 for intracellular adhesion molecule 1 (ICAM-1) *via* flow cytometry (Figure 1B). MSCs were transduced at passage 5 with either short hairpin BDNF RNA (shRNA; a form of siRNA), scrambled shRNA, or were not transduced and were cultured to passage 11–13 before being used to prepare CCM, containing the secretome of MSCs.

### Transduction and Modulation of CCM

H9C2 (ATCC) cells were maintained in Dulbecco’s modified Eagle’s low glucose (DMEM) media supplemented with 10% and 12.5% fetal bovine serum (FBS) respectively, and 1% anti-antimycotic (ThermoFisher/Gibco, Carlsbad, CA, USA, 15240-062). 293-FT (ATCC) cells were maintained in DMEM high glucose (ThermoFisher/Gibco, #11965-084) with 10% FBS, 1% MEM NEAA (ThermoFisher/Gibco, 11140-050), 1% L-Glutamine (media core 527-45) 1% anti-anti, 1% 100 mM Napyruvate Solution with or without 1% Geneticin (ThermoFisher/Gibco, #11811-023).

Six shRNA sequences were selected for initial testing to determine which would decrease BDNF expression. Six different lentiviruses were produced from co-transduced 293-FT cells with the vector pLKO.1 (Addgene, Watertown, MA, USA) with one of six shRNAs against BDNF mRNA with packaging plasmids (pMDL, pRev, and pVSVG, Invitrogen, Carlsbad, CA, USA) according to the manufacturer’s instructions. Forty-eight hours after transduction, the medium from the 293FT cells was collected and concentrated by 20,000 rpm ultracentrifugation for 2 h. The concentrated supernatant was used to transduce H9C2 cells as a test case and 4 μg/ml of puromycin was added 48 h post-transduction. Cells resistant to puromycin were selected and knockdown purity was detected by ELISA. The sequence that produced the greatest decrease in BDNF expression (5’ccggGGCGGTTCATAAGGATAGACActcgagTGTCTATCCTTATGAACCGCCtttttg3’) was chosen for the experiment and a scramble of this sequence (5’ccggGACAGAATGCGATCGGAGTTActcgagTAACTCCGATCGCATTCTGTCtttttg3’) was chosen to serve as a transduction control.

Rat MSCs were transduced at Passage 5 with the BDNF KD or scrambled lentivirus (Figure 1B). Forty-eight hours after transduction, 4 μg/ml puromycin was added to the media to select transduced cells for the first 10 days. The concentration of puromycin was reduced to 2 μg/ml in the medium to maintain the selection. Transduced MSCs were expanded and CCM was collected at passages P11-P13 (Figure 1B). CCM BDNF levels were then detected using ELISA. CCM from naive MSCs with BDNF concentration greater than 110 pg/300 μl were selected for the High BDNF CCM group.

### Characterization of CCM With Total Protein and Differentiation Assays

The concentration of total protein of each CCM batch was estimated using a Pierce^TM^ BCA Protein Assay Kit (Thermo Fisher Scientific, #23225) with bovine serum albumin (BSA, Life Technologies, Carlsbad, CA, USA, #15561020) as a standard. Adipogenesis, chondrogenesis, and osteogenesis differentiation assays (ThermoFisher/Gibco, #A10070-1, A10071-1, and A10072-1) were done according to the manufacturer’s instructions to confirm that MSCs retained stem cell characteristics after shRNA transduction in all three MSC groups at P12-P13. After differentiation, adipocytes were stained with oil red O stain on day 14, chondrocytes were stained with toluidine blue stain on day 11, and osteocytes were stained with alizarin red S stain on day 21. Images were taken with an Olympus light microscope (Center Valley, PA, USA; model: BH2-HLSH).

### Concentrated Conditioned Medium (CCM) and Concentrated Control Media (CM)

When BDNF KD, scrambled, or naive MSCs grew close to confluent (P11-P13), regular MSC culture media was replaced with antibiotic- and serum-free DMEM media for 24 h and were concentrated 50× by 4,000 rpm centrifugation for one and half hours at 4°C using Amicon ultra-15 Centrifugal Filters (Millipore, Burlington, MA, USA, #UFC900324). By the same process, CM was produced by concentrating the serum-free DMEM media not exposed to the cells. Each dose of CCM consisted of 300 μl, which was the CCM from 1 T-75 flask of MSCs, or approximately 1.5 × 10^6^ MSCs.

### ELISA

BDNF in BDNF KD, scrambled, and high BDNF CCM was quantified using a BDNF ELISA kit (G7610, Promega Madison WI, USA) according to the manufacturer’s protocol. In brief, 50 μl of CCM and 50 μl of blocker and sample buffer were added to each well. Based on duplicate assays, results were reported as total protein of BDNF pg/injection (300 μl of CCM).

### Model Creation and Treatment With CCM or CM

All rats were anesthetized with 2% isoflurane and underwent 4 h VD after bilateral PNC to create the dual injury model of SUI, as described previously (Jiang et al., 2009b; Deng et al., 2015; Janssen et al., 2019). In brief, the pudendal nerve was isolated and crushed in the ischiorectal fossa with a Castroviejo needle holder twice, each for 30 s. The vagina was accommodated for VD with increasing sizes of bouge a boule urethral dilators (24–32 Fr). A modified 10 Fr catheter was then inserted into the vagina, and then the balloon was inflated with 3 ml water for 4 h.

Sham injured rats received incisions in the dorsal skin at the same position and length as injured rats, and the vagina was accommodated with the urethral dilators. The catheter was inserted into the vagina for 4 h, but the balloon was not inflated. All rats received two subcutaneous doses of Rimadyl: one dose immediately before surgery and the second 24 h later. All rats received two doses of CM or CCM (300 μl i.p.) 1 h and 1 week after injury or sham injury.

### Functional Testing: Urinary Function and Pudendal Nerve Sensory Nerve Testing

*In vivo* functional tests, including LPP, EUS EMG, and PNSBP, were performed as described previously (Deng et al., 2019; Balog et al., 2021). Under 2% isoflurane anesthesia, a PE-50 polyethylene catheter was inserted into the rat bladder dome and a purse-string suture was used to fix the catheter before closing the abdominal wall. The catheter was then connected to a syringe pump (5 ml/h, model 200; KD Scientific, New Hope, PA) and a pressure transducer (model PT300; Natus Neurology, Providence, RI, USA). Straight parallel bipolar platinum-iridium electrodes, 1.0 mm apart (250 μm diameter; FHC, Inc., Bowdoin, ME, USA) were placed on the surface of the EUS, located at the mid-urethra, and were connected to an amplifier (band-pass frequencies: 3 Hz–3 KHz, model P511; AC Amplifier, Natus Neurology, Middleton, WI, USA) and electrophysiological recording system (10-kHz sampling rate, Power-Lab 8/35, AD Instruments, Colorado Springs, CO, USA). The pudendal nerve sensory branch was identified and isolated at the ventral side of the pudendal canal. It was suspended over a curved, parallel bipolar platinum-iridium recording electrode (250 μm diameter wire, 0.8 mm apart, 1.0 mm hook diameter; FHC, Inc., Bowdoin, ME, USA) connected to the amplifier and electrophysiological recording system and was recorded after LPP and EUS EMG recording.

Before recording, the rats were anesthetized with urethane (1.2 g/kg i.p.) and the isoflurane anesthesia was disconnected to better preserve continence and voiding function (Cannon and Damaser, 2001). The bladder was slowly filled with room temperature saline (5 ml/h) *via* the syringe pump connected to the suprapubic bladder catheter, with rats in a supine position. For LPP with simultaneous EUS EMG recording, with the bladder approximately half full, the bladder was gently and slowly depressed while recording bladder pressure and EUS EMG. The external pressure was rapidly removed when saline leakage was visualized at the urethral meatus. LPP with simultaneous EUS EMG testing was repeated several times in each rat until at least three consistent results were obtained. After LPP and EUS EMG testing, the clitoris was brushed with gauze at a moderate speed while PNSBP was recorded. This procedure was repeated four times in each animal.

### Histology

The urethra (harvested *en bloc* with the anterior vagina) and pudendal nerves were dissected, stored at −80°C, and sectioned transversely (14 μm). Transverse urethral sections underwent immunofluorescence to assess the innervation of neuromuscular junctions (NMJ) of the EUS after injury, with primary antibodies (anti-neurofilament 68, cat #N5137, and anti-neurofilament 200, cat #N0142, both 1:400 dilution, Sigma-Aldrich, St Louis, MO, USA) and secondary antibody (Alexa Fluor 488-conjugated donkey anti-mouse IgG, cat #A21202, 1:400 dilution, ThermoFisher) to identify axons, with 4 μg/ml of tetramethylrhodamine-conjugated α bungarotoxin (Rh-α-BTX, cat #T1175; ThermoFisher) to identify acetylcholine receptors at the NMJs, and with Alexa 350-conjugated phalloidin (1:20 in PBS, cat #A22281 ThermoFisher) to identify striated muscles of the EUS. Transverse pudendal nerve sections also underwent immunofluorescence to identify axons using Neurofilament 68 and 200 as the primary antibodies as above.

### Data Analysis

*In vivo* physiological data, including LPP, EUS EMG, and PNSBP, were analyzed as described previously (Deng et al., 2019). LPP was defined as baseline pressure just prior to LPP testing subtracted from peak pressure at leakage. The mean of LPP for each animal was calculated by selecting all LPPs (3–5) performed in a given animal and determining the mean LPP. Quantitative assessment of EUS EMG and PNSBP was performed by selecting a 1-s segment of baseline and a 1-s segment at peak activity to determine the mean amplitude and firing rate of muscle and nerve activity as done previously (Jiang et al., 2009b). In brief, electrophysiological analysis was performed with an automated methodology in Matlab software (R2012B, MathWorks Natick, MA, USA). EMG recordings were digitally filtered using a 60-Hz notch filter with 2-Hz bandwidth to remove powerline interference. Paired, one-second segments from EMG recordings were extracted for each LPP recording: just before the LPP manipulation (Baseline) and at peak LPP pressure (Peak). For each paired recording, a spike crossing threshold was calculated based on the interquartile range (IQR). This statistically-calculated threshold was derived entirely from the data itself, and was used rather than a fixed threshold, to adapt for intra- and inter-animal recording differences commonly encountered in EMG analysis.

Each segment was centered on a mean value of 0, and scaled to the electrode-referred voltage by dividing by the instrumentation amplifier gain, such that segments represented the amplitude in μV as recorded at the nerve. Baseline and Peak segments were combined, and the interquartile range (IQR) of paired recordings was calculated. For spike counting, any spike in the EMG waveform with the peak magnitude exceeding the IQR was identified. Spike peak values and locations were selected where the maximum signal magnitude occurred between successive crossings of the IQR threshold. For each EMG segment pair, the mean spike amplitude for identified spikes was calculated based on magnitude (in μV), and the mean firing rate was calculated as the number of identified spikes divided by the EMG segment length (1 s). An increase in EUS EMG amplitude and firing rate at peak bladder pressure during LPP testing was calculated by subtracting values at baseline from values at peak bladder pressure for each LPP trial, as modified from our prior work (Jiang et al., 2009b; Deng et al., 2019).

Mean values of each quantitative variable from each animal were determined and used to calculate a mean and standard error (SE) for each experimental group. One-way ANOVA followed by the Student-Newman-Keuls test was used to compare LPP, EUS EMG, and PNSBP results since these data were normally distributed. *P* < 0.05 indicated a statistically significant difference between groups for all statistical tests.

Immunofluorescence was evaluated qualitatively by a blinded observer. The thickness of innervating axons, the concentration of NMJs in the EUS, and integrity of the NMJs were used to evaluate the innervation of the EUS qualitatively. Nerve fascicle density and axon morphology/organization were used to assess pudendal nerve regeneration qualitatively.

## Results

BDNF concentration in CCM was significantly decreased in BDNF KD CCM (48.2 ± 3.1 pg/300 μl) compared to both scrambled CCM (94.6 ± 8.0 pg/300 μl) and high BDNF CCM (129.0 ± 13.0 pg/300 μl, Figure 1C). The concentration of BDNF in high BDNF CCM was also significantly increased compared to scrambled CCM (Figure 1C).

**Figure 2:**
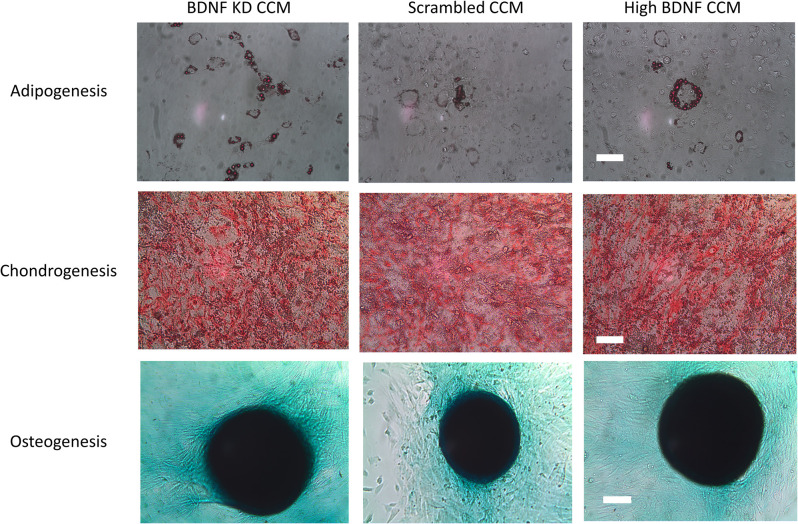
Differentiation assay. Example images of adipogenesis, chondrogenesis, and osteogenesis assays for mesenchymal stem cells (MSCs) that were either transduced with scrambled or brain-derived neurotrophin factor (BDNF) knockdown (KD) shRNA or were nontransduced controls with high BDNF levels. The scale bars for the adipogenesis and chondrogenesis assays are 100 μm. The scale bar for the osteogenesis assay is 500 μm.

MSCs transduced with either BDNF KD or scrambled siRNA demonstrated the ability to differentiate into chondrocytes, osteocytes, and adipocytes (Figure 2), as we have shown previously for these rat bone marrow derived MSCs. This demonstrated that transduction did not alter their status as MSCs (Dissaranan et al., 2014). Images were collected at the end of the differentiation, as specified in the manufactures instructions: day 14 for adipocytes, day 11 for chondrocytes, and day 21 for osteocytes. Total protein assays of CCM demonstrated no significant differences between the three different CCM groups.

**Figure 3:**
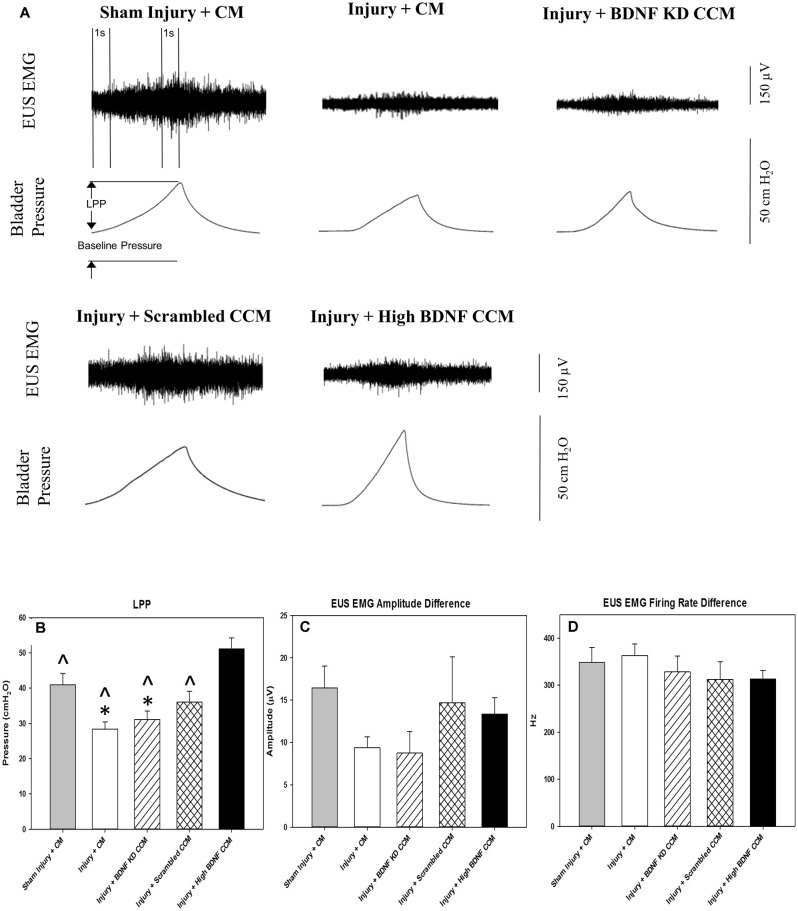
Leak Point Pressure (LPP) and External Urethral Sphincter (EUS) Electromyograph (EMG). Example of LPP and EUS EMG data for Sham Injury + Control Media (CM), Injury (Pudendal Nerve Crush + Vaginal Distension) + CM, Injury + Brain-Derived Neurotrophic Factor Knockdown (BDNF KD) Concentrated Conditioned Media (CCM), Injury + Scrambled CCM, or Injury + High BDNF CCM **(A)**. The vertical dashed lines show where 1-s EUS EMG segments were selected at baseline and peak pressure for analysis. Functional results from urinary function testing: LPP **(B)**, EUS EMG Amplitude **(C)**, and Firing Rate **(D)**. Each bar represents the mean ± standard error of the mean of 12 animals per group. ANOVA followed by a Student-Newman-Keuls *post-hoc* test was used to indicate significant differences. ^*^Indicates a statistically significant difference compared to Sham Injury + CM; ^∧^indicates a statistically significant difference compared to Injury + High BDNF CCM.

LPP examples 3 weeks after injury showed a visible decrease in LPP and EUS EMG in the injury + CM and injury + BDNF KD CCM groups compared to the sham injury + CM group (Figure 3A). When all groups were compared, LPP was significantly decreased in the injury + CM (28.3 ± 2.1 cm H_2_O) and injury + BDNF KD CCM (31.1 ± 2.5 cm H_2_O) groups compared to sham injured animals (40.9 ± 3.3 cm H_2_O, Figure 3B). LPP in the injury + scrambled CCM group (36.0 ± 3.1 cm H_2_O) was not significantly different from that of the sham injury + CM group (Figure 3B). LPP was significantly increased in the injury + high BDNF CCM group (51.1 ± 3.1 cm H_2_0) compared to all the other groups (Figure 3B).

Although the EUS EMG amplitude increase with LPP was visibly decreased in the injury + CM and injury + BDNF KD CCM groups, there were no significant differences in EUS EMG amplitude and firing rate between any of the groups: sham injury + CM (16.4 ± 2.6 μV; 348.2 ± 31.8 Hz.), injury + CM (9.4 ± 1.3 μV; 363.3 ± 53.1 Hz), injury + BDNF KD CCM (8.7 ± 2.5 μV; 328.1 ± 74.5 Hz), injury + scrambled CCM (14.7 ± 5.4 μV; 312.3 ± 84.5 Hz.), or injury + high BDNF CCM (13.4 ± 1.9 μV; 312.9 ± 42.9 Hz, Figures 3C,D).

**Figure 4:**
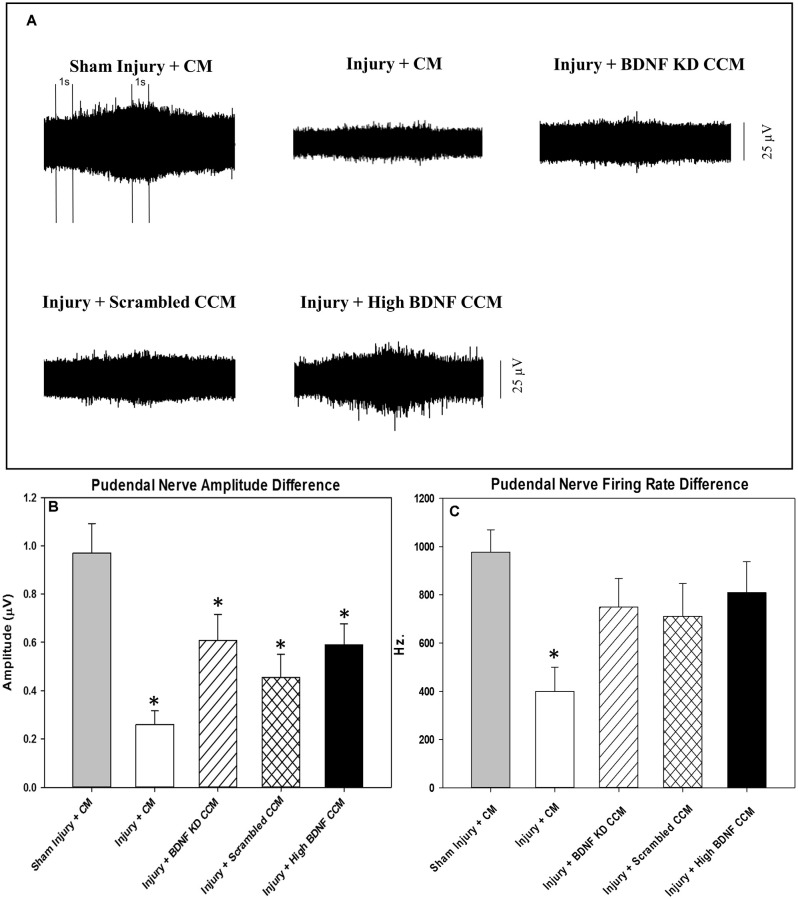
Pudendal nerve sensory branch results. Examples of sensory nerve recording from each group: Sham Injury + Control Media (CM), Injury (Pudendal Nerve Crush + Vaginal Distension) + CM, Injury + Brain-Derived Neurotrophic Factor Knockdown (BDNF KD) Concentrated Conditioned Media (CCM), Injury + Scrambled CCM, or Injury + High BDNF CCM **(A)**.The vertical dashed lines show where 1-s recording segments were selected at baseline and during brushing for analysis. Quantitative sensory nerve testing results of Amplitude **(B)** and Firing Rate **(C)**. Each bar represents the mean ± standard error of the mean of 12 animals per group. ANOVA followed by a Student-Newman-Keuls *post-hoc* test was used to indicate significant differences. ^*^Indicates a statistically significant difference compared to Sham Injury + CM.

Examples of pudendal sensory nerve functional testing showed visibly decreased activity in the injury + CM and injury + BDNF KD CCM groups compared to the sham injury + CM group (Figure 4A). Sensory nerve amplitude increased with clitoral brushing was significantly decreased in the injury + CM (0.3 ± 0.1 μV), injury + BDNF KD CCM (0.6 ± 0.1 μV), injury + scrambled (0.5 ± 0.1 μV), and injury + high BDNF CCM groups (0.6 ± 0.1 μV), compared to the sham injury + CM group (1.0 ± 0.1 μV, Figure 4B). In contrast, the sensory nerve firing rate difference was only significantly decreased in the injury + CM group (399.4 ± 100.4 Hz.) compared to the sham injury + CM group (977.4 ± 92.5 Hz, Figure 4C).

**Figure 5:**
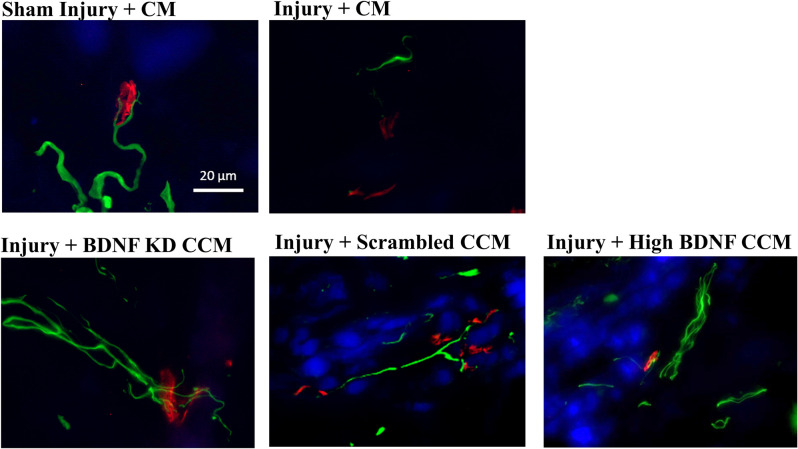
Examples of external urethral sphincter neuromuscular junction staining from each group: Sham Injury + Control Media (CM), Injury (Pudendal Nerve Crush + Vaginal Distension) + CM, Injury + Brain-Derived Neurotrophic Factor Knockdown (BDNF KD) Concentrated Conditioned Media (CCM), Injury + Scrambled CCM, or Injury + High BDNF CCM. Scale bar represents 20 μm. Green fluorescence shows axons (mouse anti-neurofilament 68 and 200), while red fluorescence indicates neuromuscular junctions (alpha-bungarotoxin stain), and blue indicates muscle (pholloid stain).

Neuromuscular junction (NMJ) immunofluorescence from EUS specimens in the injury + CM group had fewer axons and non-compact NMJ than in the sham injury + CM group, which had compact NMJs with a single innervating axon (Figure 5). NMJs of the EUS in the injury + BDNF KD CCM group demonstrated more innervating axons than in the injury + CM group but nonetheless had non-compact NMJs. The Injury + scrambled CCM group had compact NMJs innervated by a single axon, and the injury + high BDNF CCM group showed compact NMJs innervated by a single axon with many branches (Figure 5).

**Figure 6:**
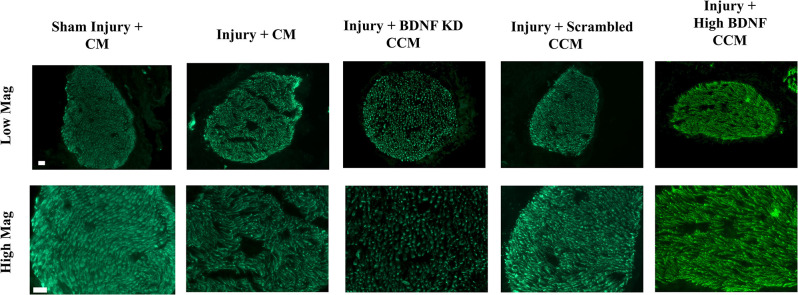
Examples of pudendal nerve sensory branch immunostaining of axons from each group: Sham Injury + Control Media (CM), Injury (Pudendal Nerve Crush + Vaginal Distension) + CM, Injury + Brain-Derived Neurotrophic Factor Knockdown (BDNF KD) Concentrated Conditioned Media (CCM), Injury + Scrambled CCM, or Injury + High BDNF CCM. The white scale bar indicates 20 μm in both top and bottom rows.

Immunofluorescence of sensory axons of the pudendal nerve in the injury + CM group had decreased axon density and organization, while the injury + BDNF KD CCM group demonstrated decreased axon density compared to the sham injury + CM group (Figure 6). In contrast, the injury + scrambled CCM and injury + high BDNF CCM groups had similar axon densities to the sham injury + CM group, demonstrating improved neuroregeneration compared to the other treated groups.

## Discussion

SUI is a common condition that affects 50% of women over the age of 60 (Snooks et al., 1990; Augoulea et al., 2017). The primary risk factor for SUI is childbirth, during which the passage of the baby through the birth canal injures both the maternal PN and the muscle it innervates, the EUS, the muscle primarily responsible for maintenance of urinary continence (Snooks et al., 1986, 1990). This creates a combinatorial neuromuscular injury in which nerve regeneration is impaired due to decreased upregulation of BDNF by the denervated EUS muscle (Pan et al., 2009; Gill et al., 2013b). MSCs and their secretome, or CCM, have been shown to improve regeneration in animal models of this dual neuromuscular injury (Deng et al., 2015; Janssen et al., 2019). To determine if BDNF is the crucial factor responsible for improved functional recovery *via* CCM treatment, we hypothesized that BDNF is necessary to facilitate functional regeneration *via* secretome from MSCs.

MSCs transduced with a BDNF KD siRNA showed a reduction in BDNF concentration of 50% compared to scrambled CCM BDNF concentration. By selecting high BDNF MSCs to create CCM for comparison, we were able to generate a simple 3-point dose response relation in functional and anatomic outcomes to the dose of BDNF in CCM. The concentration of BDNF in these three different CCM preparations were all significantly different from each other. It is worth noting that lentivirus transduction did not impair the differentiation capabilities of these rat bone marrow derived MSCs.

The differences in LPP between sham injury and injury + CM groups are similar to previously reported values, validating the dual injury model in this study (Deng et al., 2015; Jiang et al., 2018). LPP of the injury + scrambled CCM group was not significantly different from that of the sham injury or injury + CM groups. These results are similar to results presented by Deng et al. (2015) and Janssen et al. (2019) using unmanipulated CCM from rat bone marrow derived MSCs, indicating that lentivirus transduction did not impair the regenerative properties of MSC CCM. Injury + BDNF KD CCM significantly decreased LPP compared to the sham injury and injury + high BDNF CCM groups, demonstrating that BDNF is important to regeneration of the neuromuscular continence mechanism. This is supported by the outcome of our study that LPP of injury + Scrambled CCM was not significantly different from that of sham injury + CM, while LPP of injury + BDNF KD CCM was significantly decreased compared to the sham injury + CM group. This result is similar to prior work with MSCs that over-expressed proteins for angiogenesis or MSC survival given in myocardial infarction models, which found that over-expression of these proteins improved angiogenesis and regeneration (Alfaro and Young, 2012).

LPP is a global measure of the continence mechanism with several other factors contributing to urethral resistance (Jiang et al., 2011). This explains why BDNF KD CCM treatment did not result in significant differences in LPP from scrambled CCM treatment since other factors, e.g., urethral smooth muscles, could compensate for impaired EUS function (Jiang et al., 2011). This result is comparable to a study in which the BDNF regenerative pathway was inhibited after PNC, in which LPP did not change significantly but PN motor branch recordings were significantly affected by BDNF inhibition (Balog et al., 2020).

In the current study, we demonstrated that a 50% decrease in BDNF levels of MSC CCM is sufficient to slow the recovery of the neuromuscular continence mechanism. A greater decrease in CCM BDNF levels may have produced a greater reduction in LPP. Nonetheless, this study demonstrated that decreased BDNF in CCM reduced LPP 3 weeks after the injury.

LPP was the primary outcome for the study, and the study was powered for significant differences in that outcome. As a result, too few animals were studied to power a significant difference in EUS EMG outcomes, due to greater variability in these secondary outcomes. These results are similar to the study by Deng et al., which did not show a significant difference in EUS EMG between sham injury and injury + CM (Deng et al., 2015).

We showed a significant difference in PN amplitude and firing rate increase with clitoral brushing between sham injury and injury + CM, validating the dual neuromuscular injury model (Deng et al., 2019). Additionally, CCM treated groups did not have a significantly decreased firing rate compared to sham injured animals, indicating that CCM treatment facilitated axonal regeneration at least partially. While BDNF is important to sensory nerve regeneration, it is essential to motor nerve regeneration, which could explain why no PN sensory branch amplitude differences were observed between the CCM treated groups, while differences were observed between groups in LPP (Geremia et al., 2007; Balog et al., 2020). A limitation of the study is that we did not test pudendal motor branch function. However, we did investigate anatomical reinnervation of EUS NMJs by pudendal motoneurons.

NMJ staining showed healthier NMJs in all CCM treated groups compared to the injury + CM group, similar to previous publications indicating that EUS regeneration was facilitated by factors in the CCM involved in muscle regeneration (Janssen et al., 2019). These results were expected since we did not knock down other CCM components in this experiment. It is likely that by accelerating the regeneration of the EUS, this muscle began producing BDNF to facilitate PN regeneration. Animals in the injury + scrambled CCM and injury + high BDNF CCM groups showed more innervated NMJs than the injury + BDNF KD CCM group, indicating that BDNF was important for reinnervation of NMJs. Additionally, BDNF is not the only neurotrophin present in CCM that could have facilitated the regeneration of nerves, explaining axons present in the BDNF KD CCM group (Crigler et al., 2006; Oskowitz et al., 2011). However, the BDNF KD CCM group demonstrated fewer axons in PN immunofluorescence, similar to the injury + CM group, which could explain the decrease observed in LPP of the injury + BDNF KD CCM group. Martins et al. showed a decrease in axonal elongation and growth rate in neuron cell culture given CCM depleted of BDNF (~25% decrease in BDNF concentration) compared to cells given normal CCM, supporting our finding that BDNF KD CCM treatment reduced axon density (Martins et al., 2017).

One limitation of this study includes not treating rats with exogenous BDNF to determine if the effects seen are due to the BDNF alone. Previous cell culture experiments treated with MSC equivalent BDNF concentration did not see the desired effect (Martins et al., 2017). The fact that MSC levels of BDNF are much lower than the needed amount of exogenous BDNF to induce fiber elongation suggests that BDNF was not solely responsible for fiber elongation (Martins et al., 2017). Additionally, we do not report proteomic analysis of the unmanipulated, BDNF KD, or scrambled CCM to determine if there were any other changes in the CCM due to the transduction or changes in BDNF levels. However, the MSC phenotype was not affected by transduction or the changes in BDNF levels, suggesting that changes, if any, were not substantial. Another limitation is that we did not test multiple time points after injury to determine the time course of recovery or study if the EUS would increase its BDNF secretion due to the injury and treatments. In addition, quantitative histology may have added to the study but was out of the scope of the project.

Nonetheless, this study supports the theory of SUI, that BDNF is an important factor in the recovery of the neuromuscular continence mechanism (Snooks et al., 1984, 1986; Swash, 1990; Gray, 2004). Gill et al. (2013a) showed that administration of BDNF accelerated LPP recovery, supporting the results of this study. Furthermore, BDNF has been shown to be important to PN recovery since inhibiting the BDNF regenerative pathway delays functional recovery of the PN motor branch, suggesting that the decreased BDNF could result in impaired PN motor branch regeneration (Balog et al., 2020). Electrical stimulation of the PN has been shown to accelerate recovery of the continence mechanism *via* a BDNF-mediated mechanism (Jiang et al., 2018; Balog et al., 2021). The results from this study with that of the studies mentioned above, support the hypothesis that upregulation of BDNF is key for recovery from a combinatorial neuromuscular injury, in childbirth and in other neuromuscular injures.

## Conclusion

This study demonstrates that BDNF is an important contributor, but not the only factor in CCM that accelerates neuroregeneration and recovery from a dual muscle and nerve injury. Since the dose of BDNF in CCM is lower than that of exogenous BDNF, other factors in CCM also play an important role in injury repair and recovery from neuromuscular injuries. CCM may therefore provide a regenerative therapy after childbirth and other neuromuscular injuries.

## Data Availability Statement

The datasets presented in this article are not readily available because the work was funded by the Department of Veteran’s Affairs and the research was conducted at the Louis Stokes Veteran’s Affairs Medical Center and is covered under the Freedom of Information Act. Any request for information must be sent to the Department of Veteran’s Affairs and all requests will be honored. Requests to access the datasets should be directed to website: https://www.va.gov/foia/ email: vacofoiase@va.gov.

## Ethics Statement

The animal study was reviewed and approved by Louis Stokes Cleveland Veterans Affairs Medical Center Institutional Animal Care and Use Committee.

## Author Contributions

XY: participated in study design, conducting the experiment, statistical analyses, and drafting the manuscript. BB: carried out statistical analyses and drafting the manuscript. DL: participated in conducting the experiment and analyzing the data. BH: participated in conducting the experiment, carrying out statistical analyses, and drafting the manuscript. MK: participated in conducting the experiment and drafting the manuscript. HY: participated in conducting the experiment. SM: participated in analyzing the data and interpretation of results. MD: participated in study design, supervision of training and experiments, interpretation of data, and drafting the manuscript. All authors contributed to the article and approved the submitted version.

## Conflict of Interest

MD has an issued patent on MSC CCM treatment for genitourinary disorders, which is currently optioned for licensing. This study was performed with grant funding prior to optioning the intellectual property and involved no corporate input or influence. The remaining authors declare that the research was conducted in the absence of any commercial or financial relationships that could be construed as a potential conflict of interest.

## Publisher’s Note

All claims expressed in this article are solely those of the authors and do not necessarily represent those of their affiliated organizations, or those of the publisher, the editors and the reviewers. Any product that may be evaluated in this article, or claim that may be made by its manufacturer, is not guaranteed or endorsed by the publisher.

## References

[B1] Abrams P. Cardozo L. Fall M. Griffiths D. Rosier P. Ulmsten U.. (2002). The standardisation of terminology of lower urinary tract function: report from the standardisation sub-committee of the international continence society. Neurourol. Urodyn. 21, 167–178. 10.1002/nau.10052 11857671

[B2] Alfaro M. P. Young P. P. (2012). Lessons from genetically altered mesenchymal stem cells (MSCs): candidates for improved MSC-directed myocardial repair. Cell Transplant. 21, 1065–1074. 10.3727/096368911X612477 22080676

[B3] Augoulea A. Sioutis D. Rizos D. Panoulis C. Triantafyllou N. Armeni E.. (2017). Stress urinary incontinence and endogenous sex steroids in postmenopausal women. Neurourol. Urodyn. 36, 121–125. 10.1002/nau.22885 26380958

[B4] Balog B. M. Askew T. Lin D. L. Kuang M. Hanzlicek B. Damaser M. S. (2020). The pudendal nerve motor branch regenerates *via* a brain derived neurotrophic factor mediated mechanism. Exp. Neurol. 334:113438. 10.1016/j.expneurol.2020.113438 32822705

[B5] Balog B. M. Deng K. Askew T. Kuang M. Hanzlicek B. Damaser M. S. (2021). Brain derived neurotrophic factor mediates accelerated recovery of regenerative electrical stimulation in an animal model of stress urinary incontinence. Exp. Neurol. 343:113781. 10.1016/j.expneurol.2021.113781 34102241

[B6] Barzilay R. Sadan O. Melamed E. Offen D. (2009). Comparative characterization of bone marrow-derived mesenchymal stromal cells from four different rat strains. Cytotherapy 11, 435–442. 10.1080/14653240902849796 19521891

[B7] Cannon T. W. Damaser M. S. (2001). Effects of anesthesia on cystometry and leak point pressure of the female rat. Life Sci. 69, 1193–1202. 10.1016/s0024-3205(01)01182-1 11508351

[B8] Chan K. M. Gordon T. Zochodne D. W. Power H. A. (2014). Improving peripheral nerve regeneration: from molecular mechanisms to potential therapeutic targets. Exp. Neurol. 261, 826–835. 10.1016/j.expneurol.2014.09.006 25220611

[B9] Crigler L. Robey R. C. Asawachaicharn A. Gaupp D. Phinney D. G. (2006). Human mesenchymal stem cell subpopulations express a variety of neuro-regulatory molecules and promote neuronal cell survival and neuritogenesis. Exp. Neurol. 198, 54–64. 10.1016/j.expneurol.2005.10.029 16336965

[B10] Daly D. Clarke M. Begley C. (2018). Urinary incontinence in nulliparous women before and during pregnancy: prevalence, incidence, type and risk factors. Int. Urogynecol. J. 29, 353–362. 10.1007/s00192-018-3554-1 29362836

[B11] Delancey J. O. L. Trowbridge E. R. Miller J. M. Morgan D. M. Guire K. Fenner D. E.. (2008). Stress urinary incontinence: relative importance of urethral support and urethral closure pressure. J. Urol. 179, 2286–2290. 10.1016/j.juro.2008.01.098 18423707 PMC2673985

[B12] Deng K. Balog B. M. Lin D. L. Hanzlicek B. Song Q. X. Zhu H.. (2019). Daily bilateral pudendal nerve electrical stimulation improves recovery from stress urinary incontinence. Interface Focus 9:20190020. 10.1098/rsfs.2019.0020 31263536 PMC6597514

[B13] Deng K. Lin D. L. L. Hanzlicek B. Balog B. M. Penn M. S. Kiedrowski M. J.. (2015). Mesenchymal stem cells and their secretome partially restore nerve and urethral function in a dual muscle and nerve injury stress urinary incontinence model. Am. J. Physiol. Renal Physiol. 308, F92–F100. 10.1152/ajprenal.00510.2014 25377914 PMC6880193

[B14] Dissaranan C. Cruz M. A. Kiedrowski M. J. Balog B. M. Gill B. C. Penn M. S.. (2014). Rat mesenchymal stem cell secretome promotes elastogenesis and facilitates recovery from simulated childbirth injury. Cell Transplant. 23, 1395–1406. 10.3727/096368913X670921 23866688 PMC4464671

[B15] Eftekhar T. Hajibaratali B. Ramezanzadeh F. Shariat M. (2006). Postpartum evaluation of stress urinary incontinence among primiparas. Int. J. Gynaecol. Obstet. 94, 114–118. 10.1016/j.ijgo.2006.04.042 16846603

[B16] Geremia N. M. Gordon T. Brushart T. M. Al-Majed A. A. Verge V. M. K. (2007). Electrical stimulation promotes sensory neuron regeneration and growth-associated gene expression. Exp. Neurol. 205, 347–359. 10.1016/j.expneurol.2007.01.040 17428474

[B17] Gill B. C. Balog B. M. Dissaranan C. Jiang H. H. Steward J. B. Lin D. L.. (2013a). Neurotrophin therapy improves recovery of the neuromuscular continence mechanism following simulated birth injury in rats. Neurourol. Urodyn. 32, 82–87. 10.1002/nau.22264 22581583 PMC3419785

[B18] Gill B. C. Damaser M. S. Vasavada S. P. Goldman H. B. (2013b). Stress incontinence in the era of regenerative medicine: reviewing the importance of the pudendal nerve. J. Urol. 190, 22–28. 10.1016/j.juro.2013.01.082 23376143 PMC4158918

[B19] Gray M. (2004). Stress urinary incontinence in women. J. Am. Acad. Nurse Pract. 16, 188–197. 10.1111/j.1745-7599.2004.tb00441.x 15193021

[B20] Janssen K. Lin D. L. Hanzlicek B. Deng K. Balog B. M. van der Vaart C. H.. (2019). Multiple doses of stem cells maintain urethral function in a model of neuromuscular injury resulting in stress urinary incontinence. Am. J. Physiol. Renal Physiol. 317, F1047–F1057. 10.1152/ajprenal.00173.2019 31411077 PMC6880197

[B25] Jiang H.-H. Gill B. C. Dissaranan C. Zutshi M. Balog B. M. Lin D.. (2013). Effects of acute selective pudendal nerve electrical stimulation after simulated childbirth injury. Am. J. Physiol. Renal Physiol. 304, F239–F247. 10.1152/ajprenal.00235.2012 23152293 PMC3566519

[B21] Jiang H.-H. Pan H. Q. Gustilo-Ashby M. A. Gill B. Glaab J. Zaszczurynski P.. (2009a). Dual simulated childbirth injuries result in slowed recovery of pudendal nerve and urethral function. Neurourol. Urodyn. 28, 229–235. 10.1002/nau.20632 18973146 PMC2661359

[B22] Jiang H.-H. Gustilo-Ashby A. M. Salcedo L. B. Pan H. Q. Sypert D. F. Butler R. S.. (2009b). Electrophysiological function during voiding after simulated childbirth injuries. Exp. Neurol. 215, 342–348. 10.1016/j.expneurol.2008.10.024 19056383 PMC2721825

[B23] Jiang H.-H. Salcedo L. B. Damaser M. S. (2011). Quantification of neurological and other contributors to continence in female rats. Brain Res. 1382, 198–205. 10.1016/j.brainres.2011.01.094 21295013 PMC3082282

[B24] Jiang H.-H. Song Q. I. Gill B. C. Balog B. M. Juarez R. Cruz Y.. (2018). Electrical stimulation of the pudendal nerve promotes neuroregeneration and functional recovery from stress urinary incontinence in a rat model. Am. J. Physiol. Renal Physiol. 315, F1555–F1564. 10.1152/ajprenal.00431.2017 30132345 PMC6336991

[B26] Karsy M. Watkins R. Jensen M. R. Guan J. Brock A. A. Mahan M. A. (2019). Trends and cost analysis of upper extremity nerve injury using the national (nationwide) inpatient sample. World Neurosurg. 123, e488–e500. 10.1016/j.wneu.2018.11.192 30502477

[B27] Martins L. F. Costa R. O. Pedro J. R. Aguiar P. Se S. C. Fabio G. (2017). Mesenchymal stem cells secretome-induced axonal outgrowth is mediated by BDNF. Sci. Rep. 7:4153. 10.1038/s41598-017-03592-1 28646200 PMC5482809

[B28] Meyer S. de Grandi P. Kuntzer T. Hurlimann P. Schmidt N. (1993). Birth trauma: its effect on the urine continence mechanisms. Gynakol. Geburtshilfliche Rundsch. 33, 236–242. 10.1159/000272115 8130660

[B29] Mias C. Lairez O. Trouche E. Roncalli J. Calise D. Seguelas M. H.. (2009). Mesenchymal stem cells promote matrix metalloproteinase secretion by cardiac fibroblasts and reduce cardiac ventricular fibrosis after myocardial infarction. Stem Cells 27, 2734–2743. 10.1002/stem.169 19591227

[B30] Ng K. Cheung R. Y. Lee L. L. Chung T. K. Chan S. S. (2017). An observational follow-up study on pelvic floor disorders to 3-5 years after delivery. Int. Urogynecol. J. 28, 1393–1399. 10.1007/s00192-017-3281-z 28197646

[B31] Noble J. Munro C. A. Prasad V. S. Midha R. (1998). Analysis of upper and lower extremity peripheral nerve injuries in a population of patients with multiple injuries. J. Trauma 45, 116–122. 10.1097/00005373-199807000-00025 9680023

[B32] Oskowitz A. McFerrin H. Gutschow M. Carter M. L. Pochampally R. (2011). Serum-deprived human multipotent mesenchymal stromal cells (MSCs) are highly angiogenic. Stem Cell Res. 6, 215–225. 10.1016/j.scr.2011.01.004 21421339 PMC4920087

[B33] Pan H. Q. Kerns J. M. Lin D. L. Sypert D. Steward J. Hoover C. R. V.. (2009). Dual simulated childbirth injury delays anatomic recovery. Am. J. Physiol. Renal Physiol. 296, F277–F283. 10.1152/ajprenal.90602.2008 19091786 PMC2643865

[B34] Penn M. S. (2012). Are stem cells the teacher or the student? Curr. Opin. Organ Transplant. 17, 663–669. 10.1097/MOT.0b013e32835a5aad 23080068

[B35] Peter S. Evans C. Ow S. Y. Scutt A. M. Wright P. C. Biggs C. A. (2012). Proteomic analysis of the impact of static culturing on the expansion of rat bone marrow mesenchymal stem cells. Biotechnol. Lett. 34, 1589–1596. 10.1007/s10529-012-0935-2 22566207

[B36] Salam R. A. Mansoor T. Mallick D. Lassi Z. S. Das J. K. Bhutta Z. A. (2014). Essential childbirth and postnatal interventions for improved maternal and neonatal health. Reprod. Health 11:S3. 10.1186/1742-4755-11-S1-S3 25177795 PMC4145857

[B37] Snooks S. J. Barnes P. R. Swash M. (1984). Damage to the innervation of the voluntary anal and periurethral sphincter musculature in incontinence: an electrophysiological study. J. Neurol. Neurosurg. Psychiatry 47, 1269–1273. 10.1136/jnnp.47.12.1269 6512547 PMC1028132

[B38] Snooks S. J. Swash M. Henry M. M. Setchell M. (1986). Risk factors in childbirth causing damage to the pelvic floor innervation. Int. J. Colorectal Dis. 1, 20–24. 10.1007/BF01648831 3598309

[B39] Snooks S. J. Swash M. Mathers S. E. Henry M. M. (1990). Effect of vaginal delivery on the pelvic floor: a 5-year follow-up. Br. J. Surg. 77, 1358–1360. 10.1002/bjs.1800771213 2276018

[B40] Swash M. (1990). The neurogenic hypothesis of stress incontinence. Ciba Found. Symp. 151, 156–170. 10.1002/9780470513941.ch9 2226058

[B41] Taylor C. A. Braza D. Rice J. B. Dillingham T. (2008). The incidence of peripheral nerve injury in extremity trauma. Am. J. Phys. Med. Rehabil. 87, 381–385. 10.1097/PHM.0b013e31815e6370 18334923

[B42] Tran C. Damaser M. S. (2015). Stem cells as drug delivery methods: application of stem cell secretome for regeneration. Adv. Drug Deliv. Rev. 82–83, 1–11. 10.1016/j.addr.2014.10.007 25451858 PMC4398586

[B43] Zhang M. Mal N. Kiedrowski M. Chacko M. Askari A. T. Popovic Z. B.. (2007). SDF-1 expression by mesenchymal stem cells results in trophic support of cardiac myocytes after myocardial infarction. FASEB J. 21, 3197–3207. 10.1096/fj.06-6558com 17496162

